# Prevalence of insomnia in shift workers: a systematic review

**DOI:** 10.5935/1984-0063.20190150

**Published:** 2021

**Authors:** Renata Silva Brito, Cristiane Dias, Agenor Afonso Filho, Cristina Salles

**Affiliations:** 1 Escola Bahiana de Medicina e Saúde Pública, Medicina - Salvador - Bahia - Brazil.; 2 Clinos Clínica de Neurologia e Otorrinolaringologia, Medicina - Santo Antônio de Jesus - Bahia - Brazil.

**Keywords:** Sleep Initiation and Maintenance Disorders, Shift Work Schedule, Insomnia Prevalence, Workers

## Abstract

Insomnia is a sleep disorder of high prevalence with somatic and psychic repercussions. The present study aimed to describe the prevalence of insomnia in shift workers, as well as the associated variables: gender, age, marital status, profession and shift work schedule. A systematic review was performed using the descriptors “insomnia” AND “shift work”, in the PubMed, SciELO and LILACS databases, including studies that presented frequency of insomnia in shift workers, published between 2000 and 2020, in English or Portuguese, only in individuals over 18-years-old. Review articles, meta-analyzes, studies without socioeconomic information, articles without abstract and articles with participants who presented other comorbidities that justiﬁed presence of insomnia or pregnant women were excluded. From 480 studies identiﬁed, 5 were included in the analysis, with a total sample of 10,141 participants, of whom 4,183 were shift workers. The prevalence of insomnia in shift workers ranged from 12.8% to 76.4%, higher than estimated for general population. Moreover, a higher prevalence was observed among women and singles, and there was no signiﬁcant variation with age and profession. On the other hand, a relationship between shift work schedule and onset of insomnia still seems controversial.

## INTRODUCTION

Insomnia affects approximately 10 to 20% of the population in general, with about 50% having a chronic course1, characterized by difficulty in initiating or maintaining sleep and repercussions during wakefulness such as fatigue, decreased mood or irritability, general malaise and impaired attention and memory^1,2^. Such repercussions reflect on productivity, with impaired professional and social performance and consequences for physical and mental health of insomniacs^2^.

Symptoms such as muscle tension, palpitations or headache may be attributed to insomnia, and this in its most severe condition may be associated with increased risk of car or work accidents, psychiatric and cardiovascular disorders, including artery disease, arrhythmias and hypertension. Biological mechanisms were suggested as possible links between short sleep duration and these diseases, such as involvement of the autonomic nervous system, endothelial function, metabolic regulation, inflammation and coagulation system^3^.

Physiological, social and psychological factors can influence sleep. An example of this is the chronotype, characterized by a behavioral manifestation of the circadian rhythm, reflecting the individual’s propensity to sleep at a certain time of day^2^. The nocturnal chronotype has been shown to predispose to insomnia and insufficient sleep, which is maintained even after the control of variables such as sex, age and sleep duration^4^.

The measurable costs of insomnia range from direct expenses such as medical and drug treatment to expenses resulting from reduced productivity, increased absenteeism, accidents, hospitalizations, and medical costs for diseases secondary to insomnia.^5^ Annual spending in the United States related to insomnia is estimated to exceed $100 billion^6^ and a study conducted in the province of Quebec, Canada, estimated a total annual cost of $6.6 billion ^7^.

There are several reports in the literature of increased prevalence of sleep disorders in shift workers^8^, and for insomnia is no different. It is estimated that about 32% of night workers, 10% of daily workers and 8-26% of workers in rotating shifts suffer from shift work disorder^9^, outlined as the presence of insomnia and/or excessive sleepiness, accompanied by a reduction in total sleep time, associated with a work schedule that overlaps the usual sleeping time^2^. The mechanisms involved in this process affect both biological and social spheres. In biological sphere, there is disturbance in circadian rhythm, in melatonin and cortisol secretion, and in immunological functions. In social sphere, scale of shifts can impair balance work and social demands^10^.

This study aims to analyze the prevalence of insomnia in shift workers and association with demographic characteristics, due to high frequency of shift work in industrialized societies^10^ and the somatic and psychological repercussions of insomnia on individuals.

## MATERIAL AND METHODS

### Study design

Systematic review.

### Search strategies

The search strategy adopted was based on the electronic databases PubMed, SciELO and LILACS. The searches were performed by combination and contraction of descriptors, corresponding to “insomnia” and “shift work”. The terms obtained from Medical Subject Headings (MeSH) and Descriptors in Health Sciences (DeCS) were ‘’Sleep Initiation and Maintenance disorders’’; ‘’Insomnia’’; ‘’Shift Work Schedule’’ and ‘’Shift Work’’, resulting in the following search details: ((“sleep initiation and maintenance disorders” [MeSH Terms] OR (“sleep” [All Fields] AND “initiation” [All Fields] AND “maintenance” [All Fields] AND “disorders” [All Fields]) OR “sleep initiation and maintenance disorders” [All Fields] OR “insomnia” [All Fields]) OR (“sleep initiation and maintenance disorders” [MeSH Terms] OR (“sleep” [All Fields] AND “initiation” [All Fields] AND “maintenance” [All Fields] AND “disorders” [All Fields]) OR “sleep initiation and maintenance disorders” [All Fields])) AND ((“shift work schedule” [MeSH Terms] OR (“shift” [All Fields] AND “work” [All Fields] AND “schedule” [All Fields]) OR “shift work schedule” [All Fields]) OR (“shift” [All Fields] AND (“work” [MeSH Terms] OR “work” [All Fields])).

References in the articles identified by the search strategy were also searched manually in order to add to the study. The PRISMA protocol^11^ was used as a guide for the systematic review.

### Inclusion and exclusion criteria

The inclusion criteria were: studies addressing frequency of insomnia in shift workers; studies published from 01/01/2000 until 03/31/2020; studies in English or Portuguese; studies with participants aged 18 years or older; studies conducted only with human beings.

The exclusion criteria were: review articles or meta-analyzes; studies whose participants presented other comorbidities that justified the presence of insomnia; studies with pregnant women; studies without socioeconomic information; and articles without abstract.

### Identification and selection of studies

Two authors independently identified articles and evaluated their titles and abstracts, selecting them according to the inclusion and exclusion criteria. Articles not excluded by the title and with insufficient abstract for evaluation, were also listed for full reading. Each author individually assessed full texts for inclusion in this systematic review. In cases of disagreement, a third party was consulted. The manual search followed the same selection principle.

### Data extraction

The following data were extracted from the articles listed for review: title; author(s); year of publication; country of origin; scientific journal of publication; language; keywords; objective(s); study design; method; period of performance; inclusion and exclusion criteria; sample size; diagnostic criteria of insomnia; prevalence of insomnia in the sample; age of participants; gender of participants; marital status of participants; work schedule; and conclusions.

The quality of each study was analyzed based on Strengthening the Reporting of Observational Studies in Epidemiology (STROBE)12, with version validated for Portuguese in Brazil^13^. The items comprising STROBE are related to information that ought to be present in the title, abstract, introduction, methodology, results and discussion of scientific articles that describe observational studies. Eighteen items are common to cohort, case-control, and cross-sectional studies, and four items are specific to each of these three study designs^13^. Articles included in the systematic review were analyzed by the authors, giving a classification in relation to each item of STROBE: item fully met; partially met; or the fulfillment of the item was not clear. Articles that met the quality criteria in this systematic review obtained at least 11 items fully or partially met.

## RESULTS

**Figure 1:**
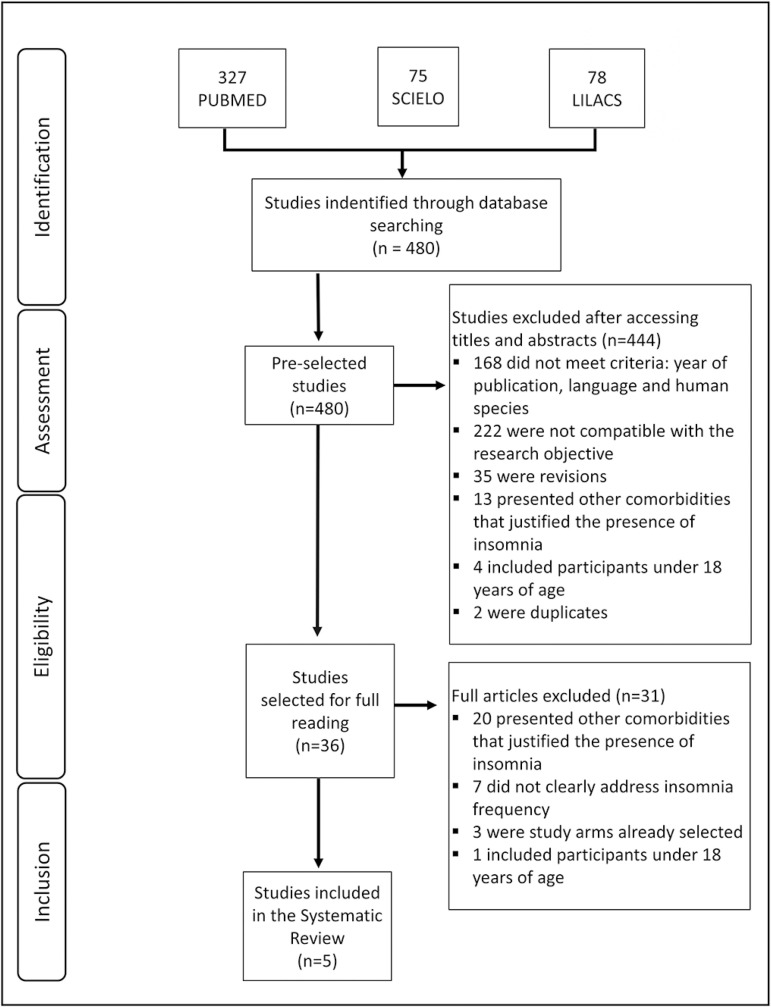
Flowchart of the procedure for selecting the studies.

Applying the combination and contraction of descriptors of Medical Subject Headings (MeSH) and Health Sciences Descriptors (DeCS), corresponding to “insomnia” and “shift work”, a total of 480 studies was obtained in the PubMed, SciELO and LILACS databases. Of these studies, 444 were excluded because they did not meet the inclusion criteria, with 36 articles remaining. After full reading, 31 were discarded due to non-conformities with the inclusion criteria. At the end, five studies were selected. A manual search in the references was performed, with a total of 207 articles, none eligible for this systematic review (Figure 1).

**Figure 2:**
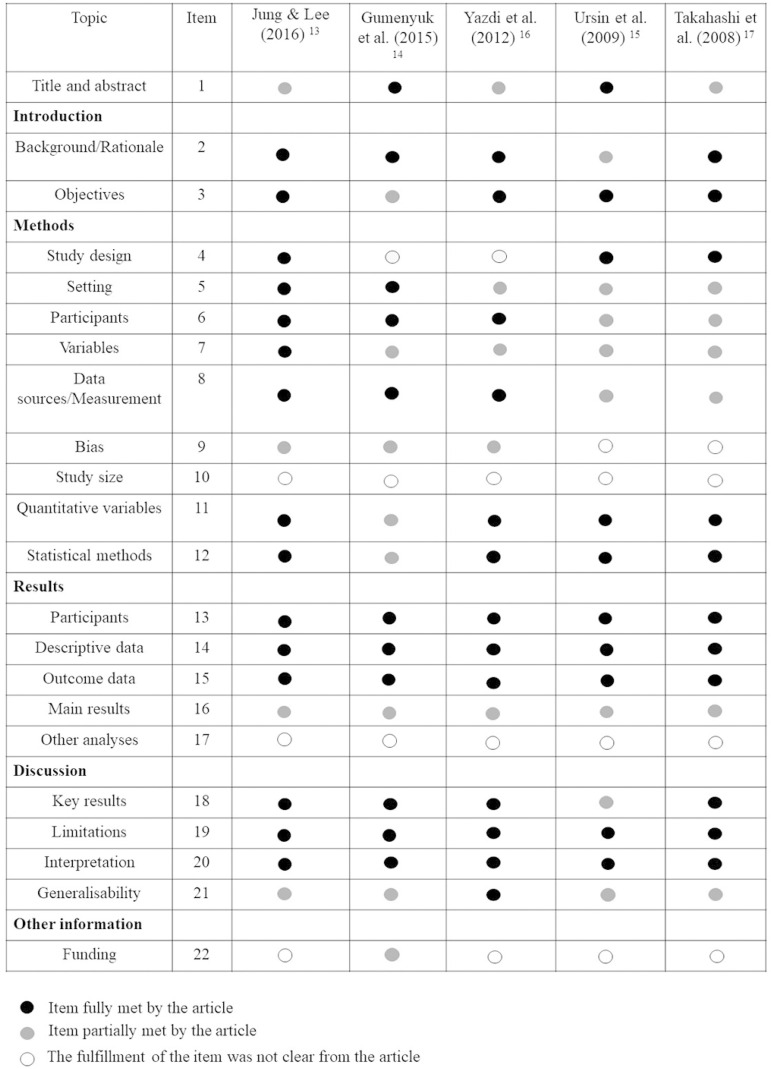
Quality assessment of selected studies according to the essential items of STROBE initiative^13^.

Regarding the quality of the selected articles, none of the studies complied with all the items proposed by STROBE, as detailed in Figure 2. The articles with the best performance were the studies of Jung and Lee (2016)14 and Gumenyuk et al.15, and none of the studies described the sample calculation and other analyses performed (items 10 and 17 of STROBE, respectively).

The selected articles had different designs: 4 descriptive cross-sectional studies and 1 observation and field study (Table 1).

**Table 1:** General characteristics of the selected studies, ordered by year of publication.

Authors (year)	Journal of publication	Country of origin	Study design	Sample (n)	Prevalence of insomnia	Diagnostic criterion	Participants' occupation
Jung & Lee (2016)^14^	Shift work and health	South Korea	Cross-sectional descriptive	1.431	1.094 (76.4%)	Insomnia Severity Index	Nurses
Gumenyuk et al. (2015)^15^	Sleep	United States	Observational laboratory and field study	37	23 (62.2%)	Insomnia Severity Index	Industrial and healthcare workers
Yazdi et al. (2012)^17^	Work	Iran	Cross-sectional descriptive	160	69 (43,1%)	Pittsburg Sleep Quality and Insomnia Severity Index	Female nurses
Ursin et al. (2009)^16^	Scand J Work Environ Health	Norway	Cross-sectional descriptive	7.782 (2.046 shift workers)	262 (12,8%)	Karolinska Sleep Questionnaire and Frequency Scale	Diverse*
Takahashi et al. (2008)^18^	Applied Ergonomics	Japan	Cross-sectional descriptive	731 (509 shift workers)	207 (40,6%)	Questionnaire about sleep problems	Nursing home caregivers

* Legislators, senior officials, managers, service providers (health personnel, teachers, police), primary occupations (farmers, fishery workers), craft and related workers, construction workers, plant and machine operators and drivers.

**Figure 3:**
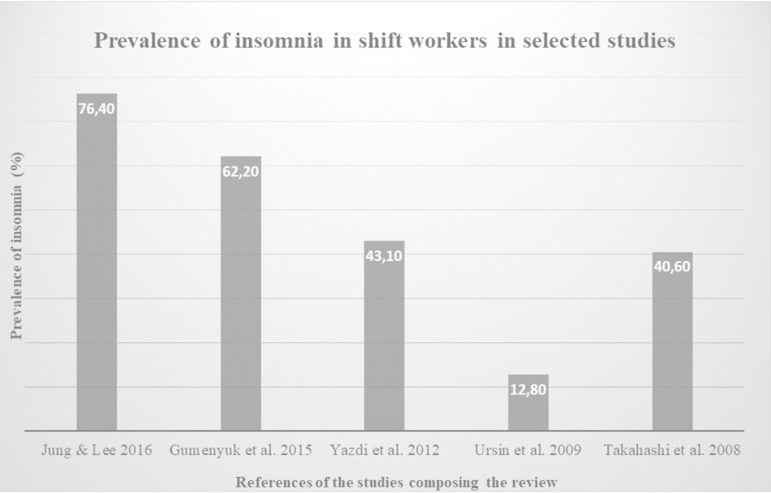
Graph of the prevalence of insomnia in shift workers in selected studies.

In the present systematic review, the added samples of the five studies resulted in a total number of 10,141 participants, 4,183 of whom were shift workers. The prevalence of insomnia in shift workers surveyed in the studies ranged from 12.8%16 to 76.4%14 (Figure 3).

Jung and Lee^14^ conducted a cross-sectional descriptive study with the aim of investigating work-related and health-related demographic factors relevant to dyspepsia and insomnia in shift nurses in South Korea. For this purpose, the sample was comprised of 1,431 nurses who worked rotating shifts with 4.9-night shifts per month and the Insomnia Severity Index (ISI) was applied to identify workers with insomnia.

In this study, Jung and Lee^14^ identified a prevalence of 76.4% of insomnia in the sample studied. Among participants who had insomnia, the proportion of single people was higher (82% vs. 74%, *p*<0.01) and they had more night shifts per month (5.2 days/month *vs*. 4.7 days/month *p*<0.001), as well as higher work-related stress score by Korean Occupational Stress Scale (KOSS) than those without insomnia (51 points vs. 49 points, *p*<0.001). The authors also observed in this study that participants with insomnia performed less moderate intensity physical activity (1.5 days/week *vs*. 1.8 days/week, *p*<0.05) and fed more irregularly than those without the condition (2.0 points *vs*. 2.5 points, *p*<0.001), which was surveyed using a 5-point score (1 = very irregular to 5 = very regular)^14^.

Gumenyuk et al.^15^ analyzed a sample of 37 night workers from health institutions and industries in order to assess insomnia symptoms in phenotypes: sleepy insomniacs and alert insomniacs. The Insomnia Severity Index was used as a diagnostic criterion for insomnia and the Epworth Sleepiness Scale was used as a diagnostic criterion for sleepiness. A prevalence of 62.2% of insomnia was identified in the sample, with approximately half corresponding to the phenotype of insomniacs without daytime sleepiness and the other half corresponding to sleepy insomniacs. In this study, objective sleepiness was also evaluated through the multiple sleep latency test (MSLT); and brain activity was measured using the N1 event-related potential. In the results, the study pointed out those insomniacs without excessive sleepiness showed greater impairment by the Insomnia Severity Index in nighttime sleep, than those who reported excessive sleepiness^15^.

Yazdi et al.^17^ conducted a descriptive cross-sectional study with the objective of assessing the relationship between morningness and eveningness chronotypes, sleep quality and insomnia in female shift worker nurses, and a sample of 160 nurses working in Iranian university hospitals was recruited, 15% of whom were permanent night workers, 45% slow rotating shift workers, and 40% fast rotating shift workers. The nurses completed the Horne and Ostberg Questionnaire to evaluate chronotypes distribution, the Pittsburg Sleep Quality Index and the Insomnia Severity Index (ISI) to measure sleep quality and insomnia, respectively. A prevalence of 43.1% of insomnia was identified in the sample. It was illustrated that nurses’ sleep time preference (chronotype) was associated with sleep quality, since evening chronotype nurses had higher sleep latency compared to morning chronotype nurses. However, shift schedule and shift patterns did not show association with nurses’ sleep quality^17^.

Ursin et al.^16^ conducted a population-based descriptive cross-sectional study in order to examine the relationship among occupation, sleep duration, sleepiness, insufficient sleep and insomnia in shift and day workers. For this purpose, a sample of 7,782 workers from diverse occupations was used, 26.3% of whom were shift workers. This study included night and on watch workers. The Karolinska Sleep Questionnaire was used to assess sleep, and for the diagnosis of insomnia was used a four point scale (“never or few times a year”, “1-2 times per month”, “about once per week” and “more than once per week”), and those who reported having episodes at least once a week were considered insomniacs^16^. They found in their study prevalence of 11.4% of insomnia in the total sample and 12.8% in the sample portion corresponding to shift workers. It was observed in this study that the effects of occupation type on sleep duration are independent of the effects of shift work. There was also no difference in the prevalence of insomnia among the diverse occupations. In addition, shift workers reported an average sleep time about 15 minutes less than day workers^16^.

Takahashi et al.^18^ conducted a cross-sectional descriptive study to evaluate the relationship between work schedule and sleep disorders in nursing home caregivers in Japan. A sample of 731 individuals was selected, 509 of whom were shift workers, subdivided into three groups: two-shift rotating workers (71.7%); three-shift rotating workers (13%); and workers in other systems - night shifts and on-call workers - (15.3%). Participants answered a questionnaire about working conditions, sleep disorders, health, lifestyle and demographic factors. Workers who declared at least one of the following symptoms were considered as insomniac: difficulty in initiating sleep (more than 30 minutes to initiate sleep); nocturnal awakenings; or waking up before desired time in the morning. The last two symptoms with a minimum frequency of 3 times a week18.

In this study, Takahashi et al.^18^ identified a 40.6% prevalence of insomnia among shift workers and 27.6% in the segment of the sample corresponding to non-shift workers. Regarding sleep disorders among groups, significant differences were observed in prevalence of insomnia, difficulty in initiating sleep, and poor sleep quality. In particular, the rotating two-shift group reported more problems than the others, with 37.6% having difficulty in starting sleep, 43% having insomnia symptoms, and 24.9% having poor sleep quality (*p*<0.01). In this subgroup, it was observed that the number of night shifts per month ranged from 1 to 6, with approximately half of the workers having 4 night shifts per month. Through multiple logistic regressions, the study verified that sleep disorders were more prevalent among those who worked 5 or more night shifts per month^18^.

Regarding the demographic characteristics of the participants of the selected articles for this systematic review (Table 2), one of them was conducted with women only^17^. The other studies had male and female participants, with a predominance of females, with 65,8% of the sample of this review. The average age of participants ranged from 27.8 (SD 4.67)14 to 45 years^16^. Two of selected studies described the marital status of participants^14,17^, with a predominance of singles in the study by Jung and Lee14 and predominance of married people in the study by Yazdi et al.^17^.

**Table 2:** Demographic characteristics of the studied sample.

Authors (year)	Sample (n)	Mean age (SD)	Gender		Marital status		Work shift
			M	F	Single	Married	
Jung & Lee (2016)^14^	1.431	27,8 (4,67)	28 (2%)	1.403 (98%)	1.141 (80%)	290 (20%)	Rotating with 4.9-night shifts per month
Gumenyuk et al (2015)^15^.	37	35,41 (9,35)	14 (38%)	23 (62%)	NI	NI	Night work
Yazdi et al. (2012)^17^	160	31,3 (5,3)	0	160 (100%)	75 (47%)	85 (53%)	Night and rotating work
Ursin et al. (2009)^16^	7.782 (2.046 shift workers)	40 a 45	3.264 (42%)	4.518 (58%)	NI	NI	Night work and watches
Takahashi et al. (2008)^18^	731 (509 shift workers)	33,6 (11,1)	162 (22,2%)	569 (77,8%)	NI	NI	Rotating two-shift system; rotating three-shift system; other system *

Abbreviations: NI: Not informed;

* Night duty or on-call.

Jung and Lee^14^ had as limitations the study design, since the cross-sectional design is insufficient to determine a causal relationship and the convenience sampling, which requires caution when applying results to the rest of the population.

Gumenyuk et al.^15^ presented as limitation the reduction of the sample by excluding shift workers who consumed large amounts of caffeine, smoked, had a high probability pretest for sleep apnea or psychiatric conditions, or used hypnotics or other medications likely to affect sleep. This limitation allows for more accurate analysis of the relationship between insomnia and shift work, but excludes a large group from the analysis. In addition, the laboratory study was carried out only once with each worker, so it is not possible to determine whether the results found are stable or variable over several nights.

Yazdi et al.^17^ presented two main limitations in their study: nurses who participated were between 23 and 41-years-old and were all women, hence the results have practical implications only for this category of shift workers. Nurses’ sleep preference with other methods such as sleep diary and polysomnography was not examined.

Ursin et al.^16^ had as limitations: restricted age of the study sample (40 to 45-years-old) since age influences the individual’s ability to adjust to repeated night shifts; and the subjectivity of sleep data, which were reported from memory on a single occasion. Moreover, in order to research insomnia a four point scale was used (“never or a few times a year”, “1 - 2 times per month”, “about once per week” and “more than once per week”) and only those who claimed to have episodes at least once per week were included in the analysis.

Takahashi et al.^18^ presented as limitations: the evaluation method of sleep disorders, since it was supported by the participants’ self-report, without using more objective resources such as polysomnography and actigraphy; the cross-sectional study design, insufficient to establish a causal relationship; and the sample selection method, which was performed in a non-randomized manner, its representativeness being questionable, besides limiting the external validation of this study^18^.

## DISCUSSION

In this systematic review, a total sample of 4,183 shift workers was obtained, observing the prevalence of insomnia in shift workers ranging from 12.8%16 to 76.4%^14^. This result is higher than estimates of this disorder for general adult population, whose studies show that prevalence varies from 6 to 30% depending on the population studied and the diagnostic criteria employed^19,20^.

Morin et al.^19^ used a sample of 2,001 individuals from the province of Quebec (Canada), who answered a telephone survey on sleep, insomnia and their treatments. Of the total sample, 25.3% were dissatisfied with sleep; 29.9% reported insomnia symptoms; and 9.5% met the criteria for insomnia syndrome. Ohayon20 conducted a literature review from studies of insomnia prevalence that considered four definitions: insomnia symptoms; insomnia symptoms with daytime consequences; sleep dissatisfaction; and insomnia diagnosis. The first definition, based on the DSM-IV insomnia criteria (difficulty in initiating sleep, maintaining sleep, and non-restorative sleep), recognizes that about one third of general population has at least one of these criteria. The second definition shows the prevalence varies between 9% and 15% when daytime consequences of insomnia are taken into consideration. The third definition, which considers sleep dissatisfaction, represents 8-18% of general population. The latter definition, more precise and corresponding to the diagnosis, establishes a prevalence of 6% of insomnia in general population^20^. Comparing estimated prevalence of insomnia in shift workers in this systematic review with that estimated for general population, the hypothesis that there is a significant association between shift work and insomnia is reinforced.

The diagnostic criteria for insomnia were different among studies selected for this systematic review. The International Classification of Sleep Disorders III defines insomnia as a persistent difficulty with sleep initiation, duration, consolidation, or quality that occurs despite adequate opportunity and circumstances for sleep, and results in some form of daytime impairment^2^. In studies and clinical practice, this condition can be measured from validated instruments.

Jung and Lee^14^ and Gumenyuk et al.^15^ used the Insomnia Severity Index as a diagnostic criterion; Yazdi et al.^17^ used the Pittsburg Index as a criterion in addition to the Insomnia Severity Index; Ursin et al.^16^ used a four-point scale for insomnia frequency; and Takahashi et al.^18^ considered as insomniacs those participants who declared difficulty in initiating sleep, nocturnal awakenings, or waking up before desired time in the morning, in line with the definition of insomnia established by the International Classification of Sleep Disorders III^2^.

Ursin et al.^16^, in addition to the insomnia frequency scale, used the Karolinska Sleep Questionnaire. This is an instrument that was tested mainly for Scandinavian and Southeast Asian samples, with no translation into English and Portuguese. It is an eighteen-question questionnaire that aims to evaluate subjective sleep and sleepiness^21^.

Among the studies listed for this review, there was a divergence in the results found. Four of selected studies^14,15,17,18^ found a prevalence of insomnia higher than 40% in their sample, while one of the studies^16^ found a prevalence of 12.8% of insomnia in the portion of the sample corresponding to shift workers. This divergence may have occurred because the latter was a population-based study^16^, with a total sample almost six times larger than the second largest sample^14^ (7782 *vs.* 1431). Furthermore, while the other studies used validated instruments to measure insomnia, the study considered as insomniacs those workers who reported having episodes of insomnia at least once per week, using a frequency scale (“never or few times a year”, “1 - 2 times a month”, “about once per week” and “more than once per week”). Thus, the prevalence of insomnia may have been underestimated in this study due to diagnostic criteria used, and consequently, different from the others.

There is a congruence in the results when comparing prevalence of insomnia in shift workers found in this systematic review with prevalence found in other studies. Kang et al.^22^ conducted a study with a sample of more than 14,000 workers from an electronics manufacturer in South Korea and they found insomnia prevalence of 53.3% among shift workers. Mello et al.^23^ conducted a study in Brazil with a sample of 400 bus drivers of interstate transport company working in shifts and they determined a prevalence of 37.5% of insomnia.

The sample selected in this systematic review was predominantly female. This factor may have contributed to the higher prevalence of insomnia observed, since the prevalence of the disorder in general population tends to be higher among women^24,25^. The relationship between gender and insomnia may be related to risk factors for insomnia since anxiety and depression are more common in women than in men and they are associated with sleep disorders. In addition, women tend to seek health services more than men do, hence may underestimate the prevalence of the disorder in males^25^. Another important factor for the higher prevalence of insomnia among women is double shift that they often take to meet both professional and domestic demands, particularly those with children^26,27^.

The average age of participants in this review ranged from 27.8 (SD 4.67)^14^ to 45-years-old16. Studies have not reported significant differences in the prevalence of insomnia between different ages. In the literature, however, there is an increase in prevalence of the disorder with age for general population^28^, which is related to changes in sleep architecture (considered normal in the aging process) and the emergence of clinical or psychiatric disorders that affect sleep^29^.

Regarding marital status, Jung and Lee^14^ demonstrated that among participants who had insomnia, the proportion of single people was higher (82% *vs*. 74%, *p*<0.01). The other studies in this review did not observe any relationship between insomnia and marital status. Other studies also show greater involvement of singles compared to married ones^28^. This can be explained by the protective factor of marriage that is as much due to the greater material security as to social/emotional support that a marriage brings^30^.

In respect of occupation of the participants of this review, the study of Ursin et al.^16^ explored the relationship of diverse professions with development of insomnia, concluding that there are no significant differences in the prevalence of insomnia among diverse occupations and the predominant factor for the development or not of the disorder is work in shifts. This finding is corroborated by other studies. Moreno et al.^31^ using a sample of 1592 individuals divided into rural workers, industrial workers and airline pilots, demonstrated a higher prevalence of insomnia among rural workers compared to industrial workers, however, when considering shift work, the prevalence of insomnia was more significant^31^.

The influence exerted by shift work schedule, meanwhile, still seems controversial. Jung and Lee^14^ observed that insomniac participants had more night shifts per month compared to non-insomniac participants. Yazdi et al.^17^ showed that shift time and shift pattern did not demonstrate any association with sleep quality in the sample studied. Takahashi et al.^18^ observed a higher prevalence of sleep disorders in the group of two-shift rotating workers, compared to the others analyzed; and in this subgroup, this prevalence is higher among those who work five or more monthly night shifts. In addition, two shift-rotating workers have as their main feature a night shift of more than 15 hours. These data together could justify the increase in sleep disorders among these workers. In the literature, other relationships are observed. Flo et al.^8^, in a study of 1,586 nurses, observed a higher risk of night shift insomnia in three-shift rotating workers compared to permanent night shift workers, as well as a higher risk of daytime insomnia in permanent night workers compared to two or three shift rotating workers^8^.

In addition to demographic factors and work shift schedule, other characteristics of workers may influence development and severity of insomnia. In the article by Yazdi et al.^17^ it was illustrated that sleep time preference (chronotype) was associated with sleep quality, because the participants of evening chronotype had higher sleep latency compared to the participants of morning chronotype. In the study by Gumenyuk et al.^15^ showed that insomnia without excessive sleepiness had worse results in night time sleep in Insomnia Severity Index when compared to insomnia with excessive sleepiness. The MSLT showed lower latency for sleepy insomniacs than alert insomniacs, and from analysis of brain activity measured using the N1 stage-related potential, increased N1 responses were observed in the alert insomniacs group compared to the others. This data indicates alert insomniacs present cortical hyperstimulation, but sleepy insomniacs do not present. Together, these results suggest insomniac phenotype without excessive sleepiness is probably an insomniac per se precipitated by shift work, while insomniac phenotype with excessive sleepiness results only from the circadian misalignment typically attributed to shift work^15^. Due to this, alert insomniacs would be more compromised.

Some limitations of this systematic review should be considered. Heterogeneity of the studies concerning insomnia diagnostic criteria, sample characteristics and variables presented, restricted the comparative analysis among the studies. The selected articles were all cross- sectional design, which restricts the determination of causal relationship. Moreover, articles whose participants had other comorbidities that justified the presence of insomnia were excluded. This criterion, although limits the sample, also allows a more accurate analysis of the relationship between insomnia and shift work.

## CONCLUSION

The present systematic review identified high prevalence of insomnia in shift workers compared to general population. Furthermore, a higher prevalence was observed among women and single people and there was no significant variation according to age and occupation. On the other hand, the relationship between work shift time and the onset of insomnia still seems controversial. This review, therefore, suggests an important association between insomnia and shift work, with following consequences: decreased professional and social performance, and repercussions on physical and mental health, which reinforces need for early identification and intervention on potential damage to health of these workers.
